# Depleting ovarian cancer stem cells with calcitriol

**DOI:** 10.18632/oncotarget.24520

**Published:** 2018-02-16

**Authors:** Amit Kumar Srivastava, Asim Rizvi, Tiantian Cui, Chunhua Han, Ananya Banerjee, Imrana Naseem, Yanfang Zheng, Altaf A. Wani, Qi-En Wang

**Affiliations:** ^1^ Department of Radiology, College of Medicine, The Ohio State University, Columbus, OH 43210, USA; ^2^ Department of Biochemistry, Faculty of Life Sciences, The Aligarh Muslim University, Aligarh, Uttar Pradesh, 202002, India; ^3^ School of Biotechnology, KIIT University, Bhubaneswar, Odisha, 751024, India; ^4^ Oncology Center, Zhujiang Hospital, Southern Medical University, Guangzhou, Guangdong, 510282, China; ^5^ Current address: Biological Science and Technology Division, CSIR-North East Institute of Science and Technology (CSIR-NEIST), Jorhat, Assam, 785006, India

**Keywords:** calcitriol, cancer stem cells (CSCs), vitamin D receptor (VDR), Wnt pathway, ovarian cancer

## Abstract

Cancer stem cells (CSCs) represent the root of many solid tumors including ovarian cancer. Eradication of CSCs represents a novel cancer therapeutic strategy. Calcitriol, also known as 1,25-dihydroxyvitamin D_3_ [1,25(OH)_2_D_3_], is an active metabolite of vitamin D, functioning as a potent steroid hormone. Calcitriol has shown anti-tumor effects in various cancers by regulating multiple signaling pathways. It has been reported that calcitriol can regulate the properties of normal and CSCs. However, the effect of calcitriol on the ovarian cancer growth and ovarian CSCs is still unclear. Here, by using a mouse subcutaneous xenograft model generated with human ovarian cancer cells, we have demonstrated that administration of calcitriol is able to strikingly delay the tumor growth. Calcitriol treatment can also deplete the ovarian CSC population characterized by ALDH^+^ and CD44^+^CD117^+^; decrease their capacity to form sphere under the CSC culture condition, and reduce the frequency of tumor-initiating cells, as evaluated by *in vivo* limiting dilution analysis. Mechanistic investigation revealed that calcitriol depletes CSCs via the nuclear vitamin D receptor (VDR)-mediated inhibition of the Wnt pathway. Furthermore, the activation of VDR pathway is more sensitive to calcitriol in ovarian CSCs than in non-CSCs, although the expression levels of VDR are comparable. Taken together, our data indicate that calcitriol is able to deplete the ovarian CSC population by inhibiting their Wnt signaling pathway, consequently, impeding the growth of xenograft tumors.

## INTRODUCTION

Epithelial ovarian cancer (EOC) is the most lethal malignancy of all gynecologic cancers with a 5-year survival rate of only 29% in advanced stages [1]. It is estimated that in 2017, about 22,440 new cases of ovarian cancer will be diagnosed and 14,080 women will die of ovarian cancer in the United States [1]. The main treatment of EOC is cytoreductive surgery followed by platinum (Pt)-based chemotherapy [2]. Chemotherapy with Pt is initially effective for most patients. However, the majority eventually becomes refractory to Pt treatment, and around 70% of patients have tumor relapses [2]. In past few years, it has been increasingly evident that a small population of cancer cells, referred to as “cancer stem cells (CSCs)”, is the most important trigger for cancer initiation and progression [3, 4, 5]. These CSCs have been identified in leukemia and a variety of solid tumors including ovarian cancers [6, 7, 8, 9]. CSCs can be identified and isolated with different combinations of cell surface markers such as CD44, CD117, MyD88, and CD133 [10, 6, 11], as well as the activity of certain enzymes such as aldehyde dehydrogenase (ALDH) [12, 11]. CSCs can also be functionally enriched by spheroid culture [6]. Given that CSCs are responsible for drug resistance and tumor relapse, eradication of CSCs is important for achieving cure [9].

Calcitriol, also known as 1,25-dihydroxyvitamin D_3_ [1,25(OH)_2_D_3_], is a hormonally active metabolite of vitamin D, functioning as a potent steroid hormone. Apart from its classical actions of calcium homeostasis and bone mineralization, calcitriol has shown anti-tumor/anti-inflammatory effects in various cancers by modulating multiple signaling pathways involved in proliferation, apoptosis, differentiation, inflammation, invasion, angiogenesis and metastasis (Reviewed in [13, 14]). The actions of calcitriol are mediated by the nuclear vitamin D receptor (VDR), a member of the nuclear receptor superfamily [15], which heterodimerizes with the retinoid X receptor (RXR) upon calcitriol binding. This VDR-RXR complex binds vitamin D responsive elements (VDRE) in gene promoters and enhancers throughout the genome and regulates gene expression [16]. Although the anti-tumor effects of calcitriol have been extensively investigated in various *in vitro* and *in vivo* human and murine tumor models including leukemia [17], squamous cell carcinoma [18], prostate [19], breast [20], and colon cancer [21], there are only limited data in ovarian cancer. It has been shown that calcitriol is able to inhibit cell growth and induce apoptosis in the *in vitro* cultured ovarian cancer cell lines [22, 23, 24]. However, the mechanisms by which calcitriol inhibits the initiation and progression of ovarian cancer have not been completely elucidated.

Several lines of evidence have demonstrated that vitamin D and analogs play an important role in the regulation of stem cells of the prostate and the skin [25, 26, 27]. Moreover, vitamin D is a well-known inducer of the terminal differentiation of human myeloid leukemia cells into monocytes and macrophages [28], possibly via mechanisms of regulating leukemic cancer stem cells/progenitors. Recently, vitamin D and its analogs were shown to reduce the number of CSCs in breast cancer [29] and thyroid cancer [30], further supporting their potential as therapeutic agents. In this study, we determined the inhibitory effects of calcitriol on ovarian CSCs and established the possible mechanism of its action. Our findings suggest that calcitriol targets ovarian CSCs via the VDR-mediated inhibition of the Wnt pathway, and therefore displays potential therapeutic utility in ovarian cancer treatment.

## RESULTS

### Calcitriol treatment reduces the CSC population in the mouse xenograft model

**Figure 1:**
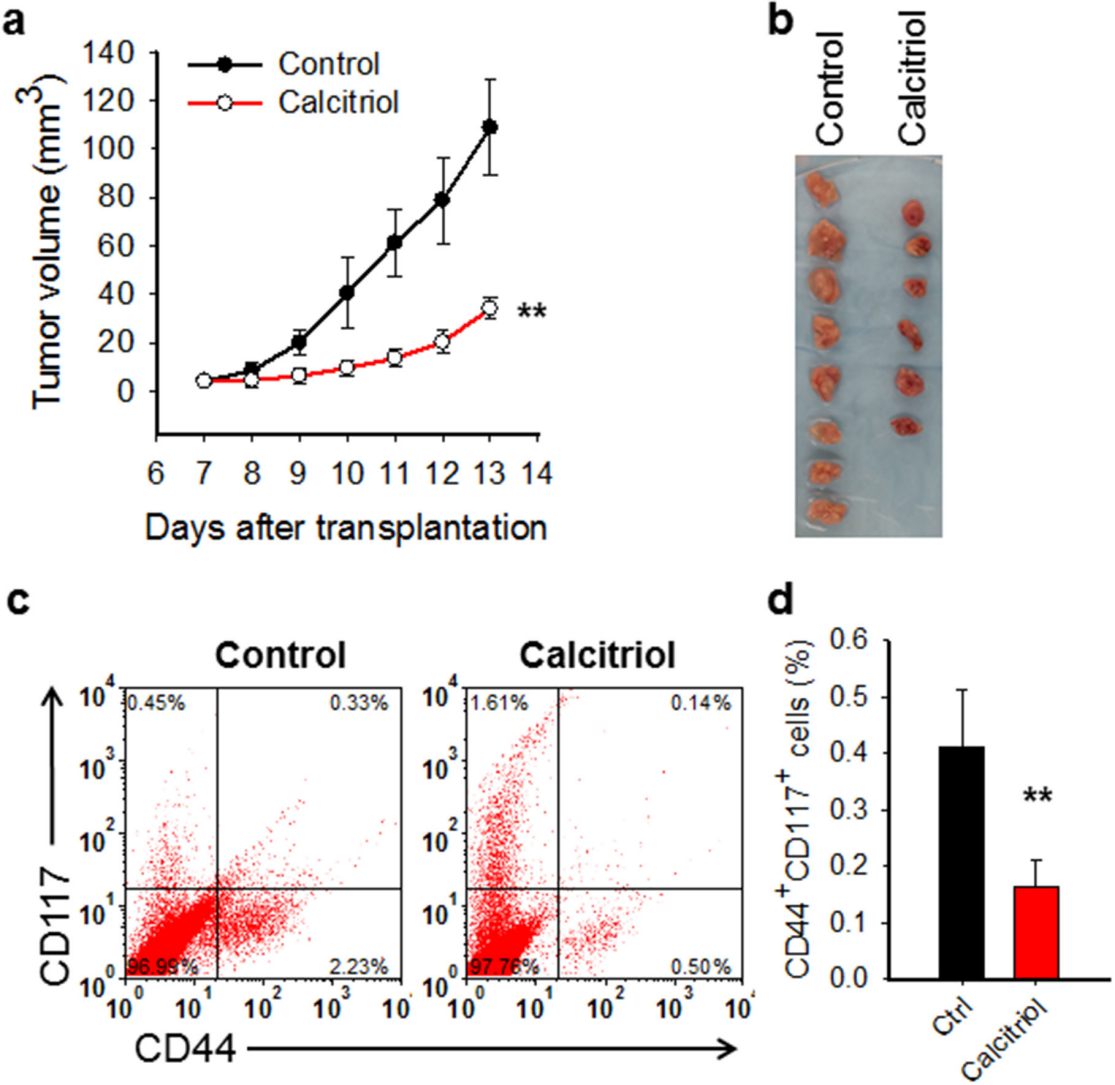
Calcitriol inhibits tumor growth and reduces CSCs in mouse ovarian xenograft model **(a, b)** Calcitriol inhibits growth of ovarian xenografts *in vivo*. The ovarian cancer cell line 2008 was injected into nude mice subcutaneously to generate xenografts. After 1 week, mice were administered with either vehicle (n=8) or calcitriol dissolved in sesame oil (n=6) i.p., 0.5 μg/Kg, once a day for 7 consecutive days. Tumor size was recorded with caliber and tumor growth curves were plotted (a) Tumor images at the harvest day were shown (b). A linear mixed effect model was used to compare the tumor growth rate between two groups. ^**^: P < 0.01. **(c, d)** Calcitriol reduces the CSC population in ovarian xenografts *in vivo*. Tumor xenografts were harvested after calcitriol treatment. Tumor cells were isolated, and the percentage of CD44^+^CD117^+^ cells in each sample was analyzed using FACS (c) and plotted (d). N = 8 for control group, and N = 6 for calcitriol group, bar: SD, ^**^: P < 0.01.

It has been reported that calcitriol is able to inhibit cell growth and induce apoptosis in the *in vitro* cultured ovarian cancer cell lines [22, 23, 24]. To determine whether calcitriol can halt ovarian tumor growth *in vivo*, we first generated subcutaneous xenograft in immunocompromised mice with an ovarian cancer cell line 2008, and then treated xenograft bearing mice with calcitriol. As shown in Figure 1a, 1b, calcitriol treatment significantly inhibited the tumor growth, providing the *in vivo* evidence for the first time that calcitriol has therapeutic promise for ovarian cancer. To further determine whether calcitriol treatment affects the CSC population in the tumor, we isolated the xenograft tumor cells after completion of calcitriol treatment, and analyzed the percentage of CD44^+^CD117^+^ cells, which have been validated to possess CSC properties [31]. As expected, *in vivo* calcitriol treatment significantly decreased the abundance of CD44^+^CD117^+^ cells in tumors (Figure 1c, 1d). Taken together, these data suggest that calcitriol is able to inhibit tumor growth in the mouse ovarian xenograft model and deplete CSCs.

### Calcitriol treatment reduces the tumorigenic CSC population in the *in vitro* cultured ovarian cancer cell line

**Figure 2:**
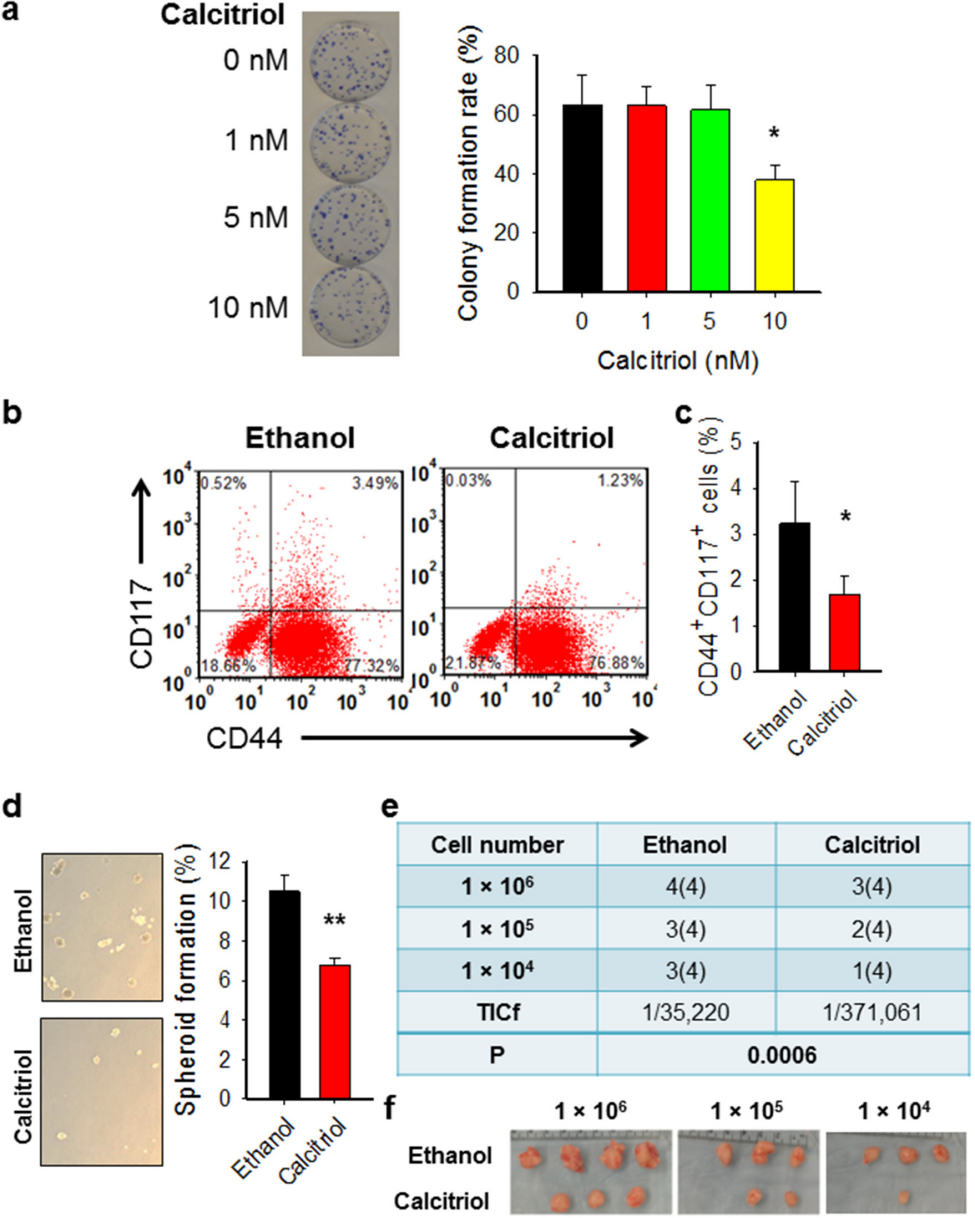
Calcitriol decreases tumorigenic CSCs in the ovarian cancer cell population **(a)** Treatment with calcitriol at 5 nM does not affect cell viability of 2008 cells. 2008 cells were treated with calcitriol at different doses, colony formation assay was conducted to determine the toxicity of calcitriol to cells. N = 3, bar: SD, ^*^: P < 0.05. **(b, c)** Calcitriol treatment reduces the CSC population in ovarian cancer cells cultured *in vitro*. 2008 cells were treated with either vehicle (ethanol) or calcitriol (5 nM) for 5 days, FACS was performed to analyze the percentage of CD44^+^CD117^+^ cells. Representative dot plots (b) and corresponding graphs (c) of FACS analysis were shown. N = 3, Bar: SD, ^**^: P < 0.01. **(d)** Calcitriol treatment reduces the sphere formation capacity of ovarian cancer cells. 2008 cells were treated with either vehicle (ethanol) or calcitriol (5 nM) for 5 days, the sphere formation capacity was determined using the sphere formation assay. N = 3, Bar: SD; ^**^: P < 0.01. **(e, f)** Calcitriol treatment reduces the TICf of ovarian cancer cells. 2008 cells were treated with either vehicle (ethanol) or calcitriol (5 nM) for 5 days, cells were injected into NOD/SCID mice subcutaneously with different cell doses (4 injections per dose). The tumor take was recorded after 4 weeks, and the TICf was calculated. Number of injection sites is in parentheses (e). The images of tumors after 4 weeks were shown (f).

To further confirm the effect of calcitriol on ovarian CSCs, *in vitro* cultured ovarian cancer cell line 2008 was treated with calcitriol at different doses. The colony formation assay has shown that calcitriol treatment at 5 nM did not affect the cell viability of bulk cancer cells (Figure 2a). We then analyzed the percentage of CD44^+^CD117^+^ cells after treatment with 5 nM of calcitriol. Our data showed that calcitriol treatment could reduce the percentage of CD44^+^CD117^+^ cells *in vitro* (Figure 2b, 2c), indicating that calcitriol is able to deplete ovarian CSCs. To further confirm the effect of calcitriol on the ovarian CSC properties, we analyzed the sphere formation capacity and the tumorigenic potential of 2008 cells after 5 nM of calcitriol treatment. As shown in Figure 2d, calcitriol treatment significantly inhibited the sphere formation capacity of 2008 cells, which is a functional indicator of CSCs. Furthermore, the xenograft assay with limiting dilution indicated that *in vitro* calcitriol treatment also decreased the frequency of tumor-initiating cells (TICf) (Figure 2e, 2f). Taken together, these data strongly indicate that calcitriol at a low dose is able to reduce the tumorigenic CSC population in the cultured ovarian cancer cells.

### Calcitriol is able to disrupt the CSC properties

**Figure 3:**
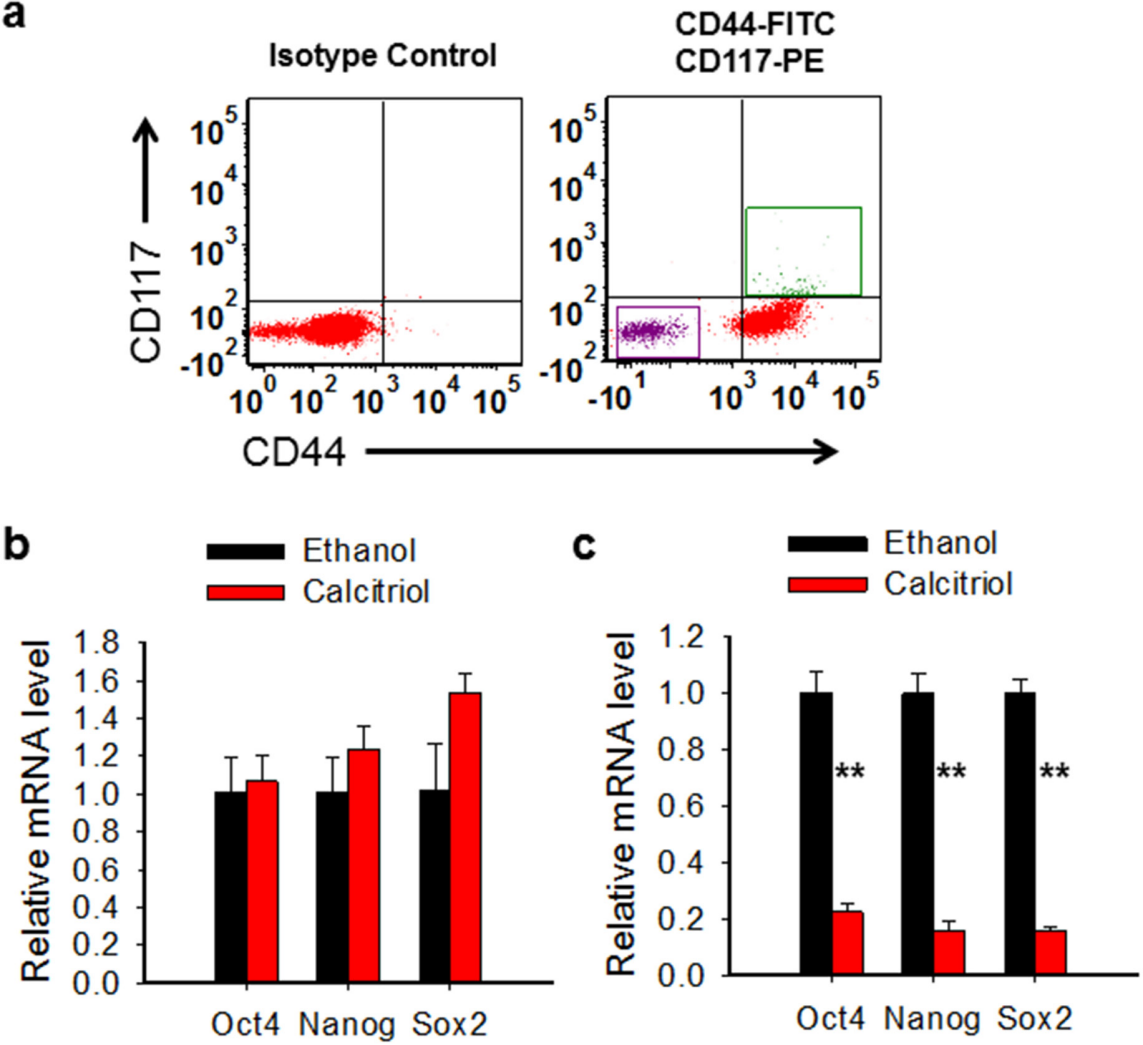
Calcitriol is able to disrupt the CSC properties **(a)** Isolation of CD44^−^CD117^−^and CD44^+^CD117^+^ cells. CD44^−^CD117^−^and CD44^+^CD117^+^ cells were sorted from 2008 cells using FACS. **(b, c)** Calcitriol treatment reduces the expression of stem cell specific genes in ovarian CSCs. CD44^−^CD117^−^ (b) and CD44^+^CD117^+^ (c) cells were treated with 5 nM calcitriol for 5 days, mRNA levels of Oct4, Nanog, and Sox2 were determined using qRT-PCR. N = 3, Bar: SD, ^**^: P < 0.01.

CSCs exhibit enhanced expression of various stem cell-specific genes, e.g., Nanog, Sox2, Oct4, which are required for the maintenance of pluripotency and the self-renewal of embryonic stem cells [32]. To determine whether calcitriol is able to inhibit the expression of these stemness genes in ovarian CSCs, we sorted CD44^+^CD117^+^ and CD44^−^CD117^−^ cells from ovarian cancer cell line 2008 using Fluorescence-activated cell sorting (FACS) (Figure 3a). These cells were treated with or without calcitriol (5 nM) for 5 days. Expression of various stem cell markers was determined using qRT-PCR analysis. As shown in Figure 3b, calcitriol treatment significantly reduced the expression of various stem cell marker genes (Sox-2, Oct-4 and Nanog) in 2008-CD44^+^CD117^+^ cells, while did not change the expression of these genes in 2008-CD44^−^CD117^−^cells (Figure 3c). Based on these results, we conclude that calcitriol treatment can disrupt the stem cell properties of ovarian CSCs.

### Ovarian CSCs exhibit enhanced VDR responses to calcitriol

**Figure 4:**
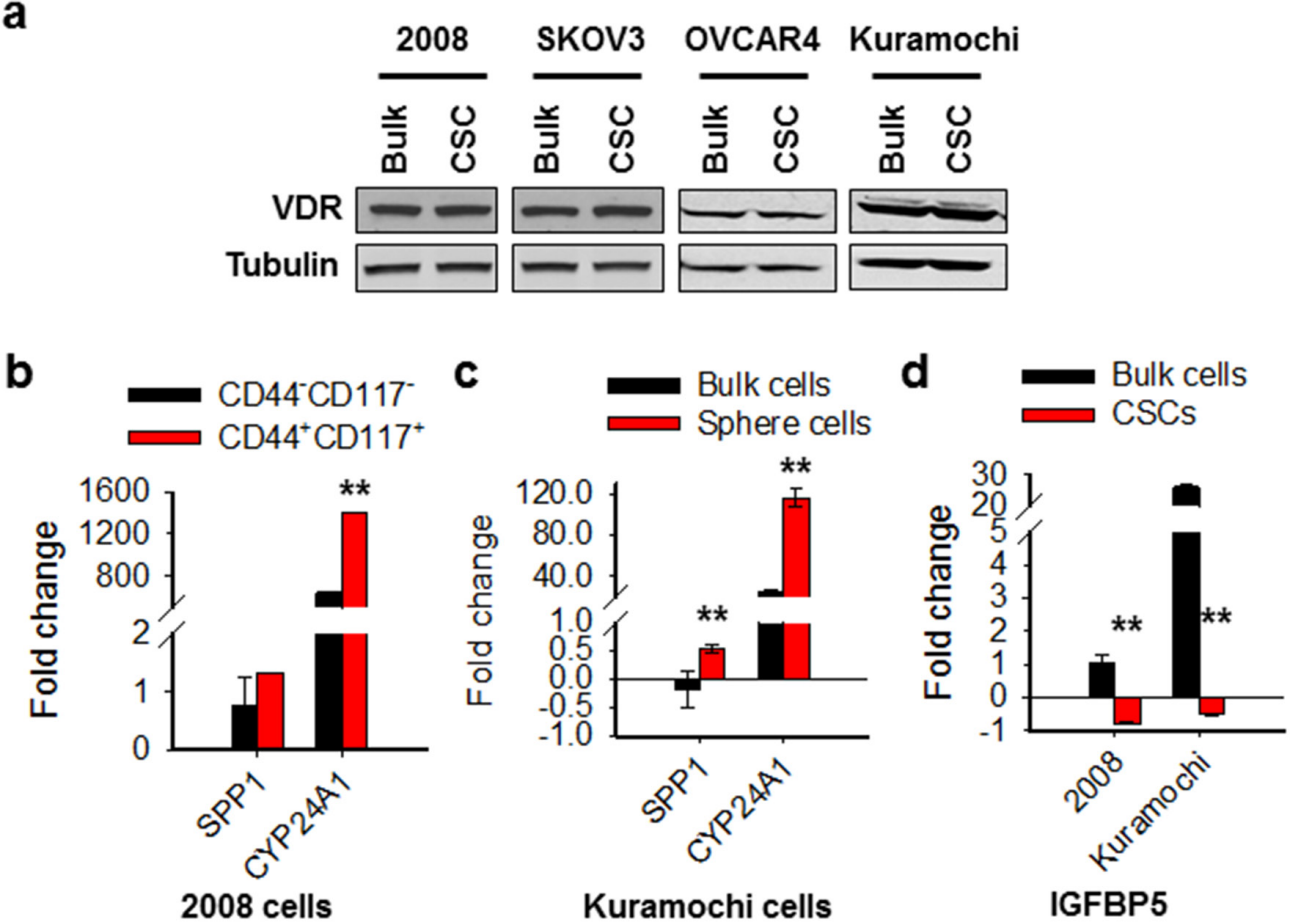
The VDR pathway responds to calcitriol differentially in ovarian CSCs and bulk cancer cells **(a)** VDR expression is comparable between CSCs and their corresponding bulk cancer cells in various ovarian cancer cell lines. CD44^+^CD117^+^ cells were isolated from 2008 cells to represent CSCs. CSCs were also enriched by sphere culturing of ovarian cancer cell line SKOV3, OVCAR4, and Kuramochi under CSC culture condition. Western blotting was carried out to detect the expression of VDR in these CSCs and their corresponding bulk cancer cells. Tubulin was also detected as a loading control. **(b, c)** Calcitriol induces more VDR target gene expression in CSCs than in bulk cancer cells. 2008-CD44^−^CD117^−^ and 2008-CD44^+^CD117^+^ cells, as well as Kuramochi bulk cancer cells and sphere cultured cells were treated with calcitriol (5 nM) for 5 days, qRT-PCR was conducted to analyze the mRNA levels of two VDR downstream genes SPP1 and CYP24A1. The gene expression fold changes compared to vehicle treatment were plotted. N = 3, Bar: SD, ^**^: P < 0.01. **(d)** Calcitriol induces VDR signaling antagonist IGFBP5 expression in bulk cancer cells, but inhibits its expression in CSCs. The mRNA level of IGFBP5 gene was determined using qRT-PCR after treatment with calcitriol as described in b and c. The gene expression fold changes compared to vehicle treatment were plotted. N = 3, Bar: SD, ^**^: P < 0.01

Calcitriol exerts most of its cellular effects via VDR [15]. Given the CSCs and bulk cancer cells exhibited different sensitivity to calcitriol treatment, we wanted to know whether the VDR signaling in CSCs and bulk cancer cells respond to calcitriol differentially. We first analyzed the expression level of VDR in CSCs and their corresponding bulk cancer cells of four ovarian cancer cell lines. No difference in VDR expression was found between CSCs and bulk cancer cells in all tested four cell lines (Figure 4a). We then treated 2008 and Kuramochi CSCs and their corresponding bulk cancer cells with calcitriol, and analyzed expression of two VDR downstream genes SPP1 and CYP24A1, which possess vitamin D-responsive element [33, 34]. As expected, both SPP1 and CYP24A1 were more induced in CSCs compared to their corresponding bulk cancer cells by the same dose of calcitriol, indicating that CSCs are more sensitive to low dose calcitriol in the activation of the VDR pathway signaling (Figure 4b, 4c). In addition, we also analyzed the expression of a VDR signaling antagonist IGFBP5 in these cells after calcitriol treatment. IGFBP5 interacts with VDR, blocks VDR/RXR heterodimerization, attenuating calcitriol-induced effects [35]. We found that calcitriol treatment increased expression of IGFBP5 in bulk cancer cells, while decreased its expression in CSCs (Figure 4d), suggesting that differential induction of IGFBP5 by calcitriol could be a reason for differential activation of VDR signaling in CSCs and bulk cancer cells. Taken together, we conclude that ovarian CSCs are more vulnerable to the VDR pathway activation following calcitriol treatment than non-CSCs.

### Calcitriol depletes CSCs in the ovarian cancer cell population through VDR-mediated inhibition of the Wnt pathway

**Figure 5:**
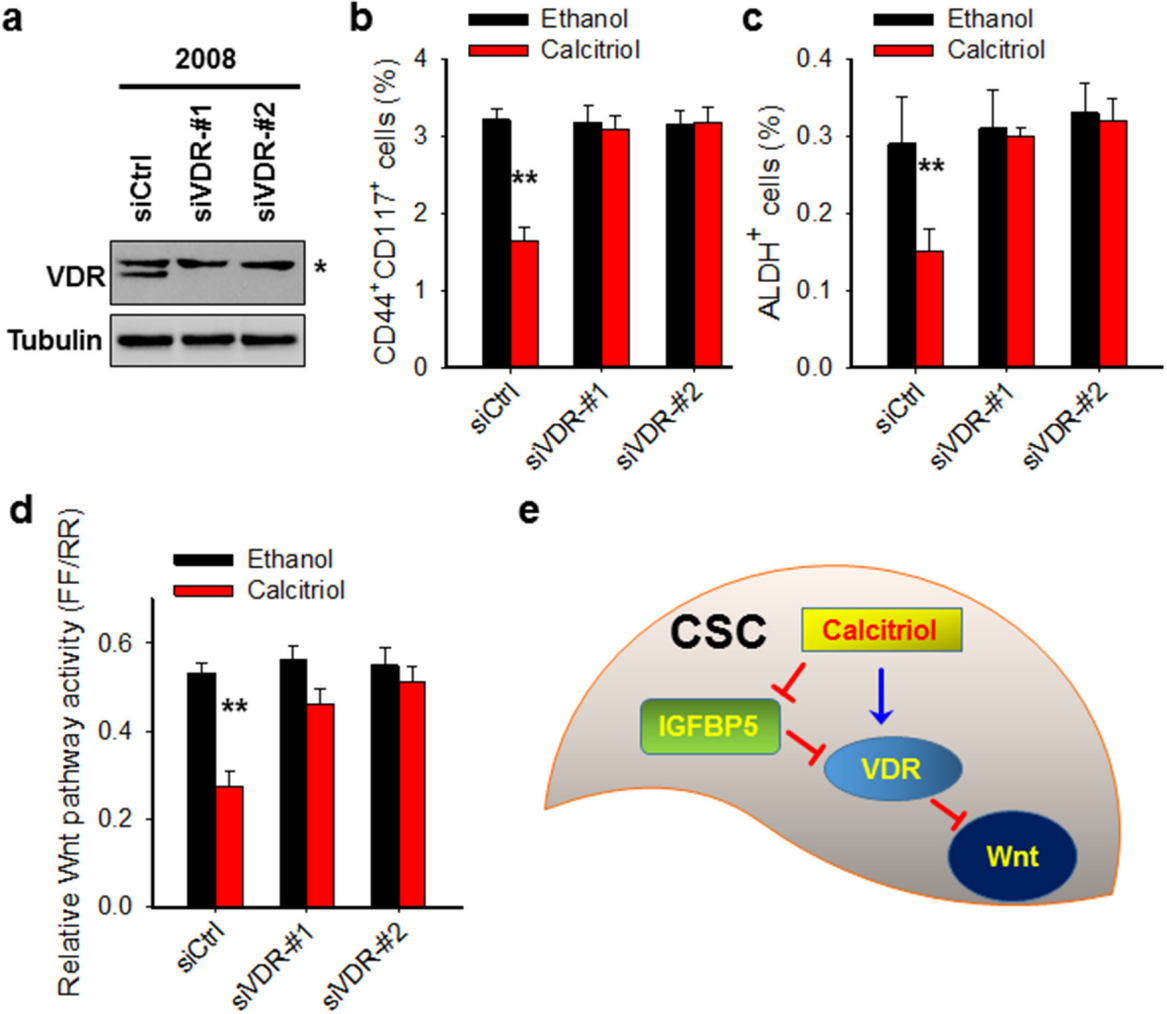
Calcitriol depletes CSCs in the ovarian cancer cell population through VDR-mediated inhibition of the Wnt pathway **(a)** VDR expression is successfully downregulated by VDR siRNA. 2008 cells were transfected with control siRNA or two different VDR siRNA for 2 days. Immunoblotting was conducted to analyze the protein level of VDR. Tubulin was detected as a loading control. ^*^: non-specific band. **(b, c)** Knockdown of VDR compromises calcitriol-induced depletion of CSCs. 2008 cells were transfected with either control or two different VDR siRNA for 24 h, treated with 5 nM calcitriol for 5 days. FACS was conducted to analyze the percentage of CD44^+^CD117^+^ cells (b) and ALDH^+^ cells (c). N = 3, Bar: SD, ^**^: P < 0.01. **(d)** Knockdown of VDR blocks calcitriol-induced inhibition of Wnt pathway activity. 2008 cells were treated same as above described, luciferase assay was conducted to determine the Wnt pathway activity. **(e)** Schematic diagram of the mechanism of calcitriol-induced depletion of CSCs. In CSCs, calcitriol treatment inhibits the expression of IGFBP5, which is an antagonist of the VDR pathway signaling. The reduced expression of IGFBP5 can promote calcitriol-induced activation of the VDR pathway signaling, which further inhibits the Wnt pathway, leading to the loss of stem cells’ properties of CSCs.

Given that the VDR pathway signaling in CSCs can be dramatically activated by calcitriol, and CSCs lose their stemness properties after calcitriol treatment, we reasoned that calcitriol may deplete CSCs via VDR signaling activation. To this end, we downregulated VDR expression in the ovarian cancer cell line 2008, treated cells with calcitriol, and determined the CSC populations characterized by CD44^+^CD117^+^ or ALDH^+^. As shown in Figure 5a-5c, calcitriol treatment significantly reduced both CD44^+^CD117^+^ and ALDH^+^ cells in this ovarian cancer cell line, while knockdown of VDR blocked calcitriol-induced decrease of these cells, indicating that the depletion of CSCs by calcitriol treatment is a VDR-dependent event.

Wnt/β-catenin signaling plays an important role in normal and cancer stem cell function [36, 37]. A series of studies have shown that the activated VDR pathway can inhibit Wnt/β-catenin signaling [38, 39]. We then confirmed the involvement of VDR signaling in the inhibition of Wnt signaling by analyzing the TCF/LEF reporter activity with or without VDR in ovarian cancer cells. Calcitriol treatment significantly reduced the TCF/LEF reporter activity in ovarian cancer cell line (Figure 5d). Downregulation of VDR by using siRNA blocked calcitriol-decreased Wnt pathway signaling activity, indicating that calcitriol depletes CSCs via the VDR-mediated inhibition of the Wnt pathway (Figure 5d). Taken together, our data indicate that calcitriol is able to deplete the ovarian CSC population by inhibiting their Wnt signaling pathway via the VDR pathway.

## DISCUSSION

Although the anti-tumor effects of calcitriol have been widely investigated in various tumors, there are only limited data in ovarian cancer. Calcitriol has been shown to inhibit cell growth and induce apoptosis in the *in vitro* cultured ovarian cancer cell lines [22, 23, 24]. Here, we demonstrated that calcitriol can also cause a strikingly delay in ovarian tumor growth in the mouse subcutaneous xenograft model. Moreover, we also showed that calcitriol is able to deplete ovarian CSCs by VDR-mediated inhibition of the Wnt pathway.

Calcitriol functions by binding to and activating the VDR [15]. The previously reported inhibition of CD44 expression in breast cancer cells by calcitriol analogue BXL0124 can be blocked by downregulation of VDR [29], suggesting that the VDR plays a critical role in the regulation of CSC population by calcitriol. In fact, our data also demonstrated that downregulation of VDR blocked calcitriol-caused reduction of ovarian CSCs characterized by CD44^+^CD117^+^ or ALDH^+^. Given that calcitriol regulates the expression of many targeted genes through VDR, we posited that the VDR pathway in ovarian CSCs responds to calcitriol in a different way from that in bulk EOC cells. In support of this hypothesis, our data demonstrated that in terms of induction of VDR downstream gene expression, calcitriol exhibited a more significant effect on ovarian CSCs than bulk cancer cells. Thus, although CSCs and their corresponding bulk cancer cells possess comparable VDR expression levels, they exhibit different responses to calcitriol in the VDR signaling activation.

During the VDR signaling activation, calcitriol promotes the heterodimerization of VDR and RXR to regulate gene expression. This heterodimerization can be blocked by the binding of IGFBP5 to VDR [35]. Our data have shown that calcitriol treatment induced expression of IGFBP5 in ovarian bulk cancer cells, but inhibited IGFBP5 expression in CSCs. IGFBP5 is a VDR downstream gene possessing VDRE [40]. Thus, it is not surprising that calcitriol treatment induces IGFBP5 expression in ovarian cancer cells. It has been reported that IGFBP5 expression is decreased during N-(4-hydroxyphenyl)retinamide-induced neuronal differentiation of human retinal pigment epithelial, and this IGFBP5 downregulation is mediated by the MAPK pathway involving decreased binding of transcription factor C/EBPβ to the IGFBP5 promoter [41]. Interestingly, calcitriol treatment can activate the ERK signaling independently of the VDR [42]. Thus, it is likely that during calcitriol-induced differentiation of ovarian CSCs, calcitriol activates the MAPK pathway in CSCs, and downregulates IGFBP5 expression. Thus, the increased IGFBP5 can further compromise calcitriol-induced activation of VDR signaling in bulk cancer cells, while the decreased IGFBP5 can promote this VDR signaling activation in CSCs, leading to differential expression of VDR target genes in CSCs and their corresponding bulk cancer cells upon calcitriol treatment.

Recent studies have shown that vitamin D and analogs are able to regulate CSC properties by affecting various signaling pathways, such as Wnt, Notch, Hedgehog and TGF-β [13]. Among these pathways, Wnt/β-catenin signaling can be inhibited by the VDR pathway [38, 39]. Calcitriol has been shown to disrupt Wnt/β-catenin signaling through multiple mechanisms. In colon cancer lines, calcitriol induces the VDR to directly bind to β-catenin and increases E-cadherin expression to sequester β-catenin at adherens junctions, which leads to a reduced Wnt pathway activation [43]. Moreover, Jeong et al [44] have shown that calcitriol significantly decreased the percentage of tumor initiating cells in mouse mammary tumors through blockage of the Wnt/β-catenin pathway. In this study, we also demonstrated that calcitriol is able to inhibit the Wnt pathway, and this effect is mediated by calcitriol-activated VDR signaling.

In summary, we have elucidated an inhibitory effect of calcitriol on ovarian CSCs. Calcitriol treatment can preferentially activate the VDR signaling pathway in CSCs, which further inhibit the Wnt pathway and disrupt the CSC's stemness, leading to a reduction of the CSC population (Figure 5e). Given that the existence of CSCs in tumors has been implicated in disease recurrence, metastasis, and therapeutic resistance, eradication of CSCs by treatment with low dose of calcitriol could be exploited for improving the efficacy of the first line therapy of ovarian cancer.

## MATERIALS AND METHODS

### Cell culture and calcitriol treatment

Human ovarian cancer cell line 2008 was kindly provided by Dr. Francois X. Claret (University of Texas, M. D. Anderson Cancer Center). The SKOV3 ovarian cancer cell line was provided by Dr. Thomas C. Hamilton (Fox Chase Cancer Center). Kuramochi and OVCAR4 cells were kindly provided by Dr. Adam Karpf (University of Nebraska). All cell lines were authenticated by DNA (short tandem repeat) profiling and maintained in Roswell Park Memorial Institute medium 1640 (RPMI 1640) supplemented with 10% (vol/vol) fetal bovine serum. The 2008-CD44^+^CD117^+^ cells, Kuramochi-spheroid, OVCAR4-spheroid, and SKOV3-spheroid cells were maintained in Ultra-Low Attachment plates in KnockOut Dulbecco's Modified Eagle Medium (DMEM)/F12 medium supplemented with 20% KnockOut Serum Replacement (Life Technologies), 20 ng/mL epidermal growth factor (EGF), and 10 ng/mL basic fibroblast growth factor (bFGF). All cells were grown at 37 °C in humidified atmosphere of 5% (vol/vol) CO_2_ in air. Calcitriol was purchased from Cayman Chemical (Ann Arbor, MI). For *in vitro* treatment, calcitriol was dissolved in ethanol. For *in vivo* treatment, calcitriol was dissolved in sesame oil.

### Flow cytometry analysis and cell sorting

Anti–CD117-PE and anti–CD44-FITC (BD Pharmingen) were used for FACS analyses. Briefly, cells were incubated with antibodies on ice for 40 min in the dark. After washing with cold PBS, cells were resuspended in 200 μL PBS and subjected to FACS analyses on a BD FACS Aria III at The Ohio State University Analytical Cytometry Shared Resource.

### Quantitative reverse transcription PCR (qRT-PCR) analysis

Total RNA was extracted using Trizol reagent (Life Technologies, Carlsbad, CA). The first strand cDNA was generated by the Reverse Transcription System (Promega, Madison, WI) in a 20 μL reaction containing 1 μg of total RNA. A 0.5 μL aliquot of cDNA was amplified by Fast SYBR Green PCR Master Mix (Life Technologies) in each 20 μL reaction. PCR reactions were run on the ABI 7900 Fast Real-Time PCR system in the OSUCCC Nucleic Acid Core Facility with the following primers: Oct4, forward, 5’- TCG CAA GCC CTC ATT TCA CC- 3’, reverse, 5’- CGA GAA GGC GAA ATC CGA AG-3’; Sox2 , forward, 5’- TCA GGA GTT GTC AAG GCA GAG-3’, reverse, 5’- GGC AGC AAA CTA CTT TCC CC-3’; Nanog, forward, 5’- GTC CCA AAG GCA AAC AAC CC-3’, reverse, 5’- TTG ACC GGG ACC TTG TCT TC-3’; ; SPP1, forward, 5’- GAG GGC TTG GTT GTC AGC-3’, reverse, 5’- CAA TTC TCA TGG TAG TGA GTT TTC C −3’; CYP24A1, forward, 5’- GGG TGA ATA TGC TTT ACC CAA A- 3’, reverse, 5’- AGA TGC GCA AAA GGA TTA ATT T −3; IGFBP5, forward, 5’-AGA GCT ACC GCG AGC AAG T- 3’, reverse, 5’-AGT AGG TCT CCT CGG CCA TC-3; GAPDH, forward, 5’-GAA GGT GAA GGT CGG AGT- 3’, reverse, 5’-GAA GAT GGT GAT GGG ATT TC-3’. The relative expression values of these genes were calculated and normalized to GAPDH in each sample and compared. The experiments were performed in triplicates.

### Immunoblotting

Whole-cell lysates were prepared by boiling cell pellets for 10 min in SDS lysis buffer [2% (wt/vol) SDS, 10% (vol/vol) Glycerol, 62 mmol/L Tris·HCl, pH 6.8, and a complete miniprotease inhibitor mixture (Roche Applied Science)]. After protein quantification, equal amounts of proteins were loaded, separated on a polyacrylamide gel, and transferred to a nitrocellulose membrane. Protein bands were immune detected with appropriate antibodies, e.g., rabbit anti-VDR (Cell Signaling Technology), and mouse anti-Tubulin (Cell Signaling Technology).

### Sphere formation assay

A total of 1,000 cells were mixed with semisolid media (MethoCult H4100, STEMCELL Technologies) containing serum-free DMEM/F12 medium supplemented with 20% KnockOut Serum Replacement, 20 ng/ mL EGF, and 10 ng/mL bFGF (Life Technologies), and seeded in 6-well Ultra-Low Attachment plates (Corning). Sphere formation was assessed 10 days after cell seeding.

### Colony formation assay

A total of 150 cells were plated into a 60-mm dish, treated with calcitriol at different doses, and cultured for 14 days to allow colony formation. Colonies were fixed with 3.7% formaldehyde, and stained with 1% methylene blue for counting.

### Small interference RNA (siRNA) transfection

siGENOME human VDR siRNA-#1 (5’-GCA ACC AAG ACU ACA AGU AdTdT-3’), VDR siRNA-#2 (5’-UCA AUG CUA UGA CCU GUG AdTdT-3’), and non-targeting control siRNA (5’-UUC UCC GAA CGU GUC ACG UdTdT-3’) were purchased from Dharmacon. SiRNA Control and siVDR (100 nM) were transfected into cells using Lipofectamine 2000 (Life Technologies).

### Wnt pathway activity detection

Baseline Wnt activity was measured by using the TCF/LEF Reporter Kit (BPS Bioscience) as previously described [45]. Briefly, cells were seeded at a density of 10,000 in a 96-well plate, transfected with either negative control or reporter plasmid according to the manufacturer's instruction. Wnt pathway activity was detected using the Dual Luciferase Assay System, normalized with Renilla, and calculated according to the TCF/LEF Reporter Kit data sheet.

### Xenograft tumor model

Nonobese diabetic/severe combined immunodeficiency and Athymic nude (NCr-nu/nu) mice (6–8 wk, female, 20–25 g body weight) were obtained from National Cancer Institute (Frederick, MD). Animals were maintained in accordance with institutional policies, and all studies were performed with approval of the Institutional Animal Care and Use Committee at the Ohio State University. To generate xenografts, 5 × 10^6^ 2008 cells were mixed (1:1) with Matrigel (BD Biosciences) and injected subcutaneously (s.c.) into the flank of each mouse. After 1 week, mice were divided into two groups minimizing weight and tumor sizes differences. Mice were administered with either vehicle (n=8) or calcitriol dissolved in sesame oil (n=6) intraperitoneally (i.p.), 25 μg/Kg, once a day for 7 consecutive days. Tumor growth was measured using calipers, and volumes were calculated based on the formula V = (a × b^2^)/2, in which *a* is the longest and *b* is the shortest diameter of the tumor. Xenograft cells were isolated after calcitriol treatment with the help of collagenase digestion and RBC lysis buffer (eBioscience). To determine the frequency of tumor-initiating cells (TICf) using the limiting dilution assay (LDA), three cell doses (1 × 10^6^, 1 × 10^5^, 1 × 10^4^) of each sample were injected s.c. into the axillas of NOD/SCID mice. Mice were monitored for up to four weeks post-injection, and the tumor number per group within this period was used to calculate the TICf using the ELDA software (http://bioinf.wehi.edu.au/software/elda/index.html) [46].

### Statistical analysis

Linear mixed effect models were used to take into account the observations from the same subject for the tumor growth studies. Tumor growth over time was compared among groups from those models. For the qRT-PCR studies, the data were first normalized to the internal control, and then t-test was used to analyze difference between groups. All results were presented as mean ± SD, with a P value < 0.05 considered as statistically significant.
